# Dynamic co‐culture metabolic models reveal the fermentation dynamics, metabolic capacities and interplays of cheese starter cultures

**DOI:** 10.1002/bit.27565

**Published:** 2020-09-28

**Authors:** Emrah Özcan, Merve Seven, Burcu Şirin, Tunahan Çakır, Emrah Nikerel, Bas Teusink, Ebru Toksoy Öner

**Affiliations:** ^1^ Systems Biology, Amsterdam Institute of Molecular and Life Sciences (AIMMS), VU Amsterdam Amsterdam The Netherlands; ^2^ Department of Bioengineering IBSB, Marmara University Istanbul Turkey; ^3^ Genetics and Bioengineering Department Yeditepe University Istanbul Turkey; ^4^ Department of Bioengineering Gebze Technical University Gebze Kocaeli Turkey

**Keywords:** lactic acid bacteria, starter cultures, genome‐scale metabolic network, co‐culture metabolic modelling

## Abstract

In this study, we have investigated the cheese starter culture as a microbial community through a question: can the metabolic behaviour of a co‐culture be explained by the characterized individual organism that constituted the co‐culture? To address this question, the dairy‐origin lactic acid bacteria *Lactococcus lactis* subsp. *cremoris*, *Lactococcus lactis* subsp. *lactis*, *Streptococcus thermophilus* and *Leuconostoc mesenteroides*, commonly used in cheese starter cultures, were grown in pure and four different co‐cultures. We used a dynamic metabolic modelling approach based on the integration of the genome‐scale metabolic networks of the involved organisms to simulate the co‐cultures. The strain‐specific kinetic parameters of dynamic models were estimated using the pure culture experiments and they were subsequently applied to co‐culture models. Biomass, carbon source, lactic acid and most of the amino acid concentration profiles simulated by the co‐culture models fit closely to the experimental results and the co‐culture models explained the mechanisms behind the dynamic microbial abundance. We then applied the co‐culture models to estimate further information on the co‐cultures that could not be obtained by the experimental method used. This includes estimation of the profile of various metabolites in the co‐culture medium such as flavour compounds produced and the individual organism level metabolic exchange flux profiles, which revealed the potential metabolic interactions between organisms in the co‐cultures.

## Graphical abstract

Co‐cultures of lactic acid bacteria were investigated using dynamic genome‐scale metabolic modelling techniques. Özcan and co‐workers estimated the metabolic capacity, microbial abundance, potential of flavour compounds production and metabolic interactions of the co‐cultures using the features of individual organisms that constituted the co‐cultures.

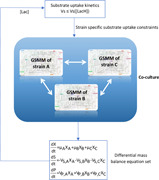

## 1 INTRODUCTION

Milk has been processed by humankind for millennia and cheese is one of the oldest fermented dairy food (Salque et al., 2013). Cheese is traditionally made either by the lactic acid bacteria (LAB) naturally present in milk or by the back‐slopping technique, which is adding a small portion of a previous batch of cheese to milk. On the other hand, in industrial cheese production, defined mixtures of purified and characterized LAB, known as starter cultures, are used to standardize the bulk production (Cogan & Hill, 1993; Leroy & De Vuyst, 2004; Powell et al., 2011). Acidification and flavour compound production are the main functions of the starter cultures in cheese making (Smid & Kleerebezem, 2014; Smit et al., 2005). Cheese starter cultures are composed of different sets of LAB for different cheese types and can be grouped as mesophilic and thermophilic starter cultures (Cogan & Hill, 1993). Mesophilic starter cultures are used in the cheese production requiring moderate temperature (~30°C) such as Dutch type cheese, and they are dominated by *Lactococcus lactis* and *Leuconostoc mesenteroides* strains (Smid et al., 2014). Thermophilic starter cultures are used in the cheese production requiring higher temperature such as Swiss and Italian cheeses and they are dominated by *L. lactis* and *Streptococcus thermophilus* strains (Cogan & Hill, 1993; Powell et al., 2011). The success of producing a cheese with desired features such as aroma and texture highly depends on the starter cultures being used. While individual strains in monoculture have been well characterized physiologically and modelled, much less is known about their behaviour when these strains are put together. Most microbial ecology approaches deal with species abundances via (meta)genomics, not with the metabolic exchange fluxes. We here address the question, whether the properties of strains in isolation can be used to predict their behaviour in co‐culture.

In this study, we have investigated dairy origin LAB in cheese starter cultures by a dynamic metabolic modelling approach based on genome‐scale metabolic networks of involved organisms. There are several genome‐scale metabolic modelling studies of dairy‐origin LAB at single‐species level (Flahaut et al., 2013; Oliveira et al., 2005; Özcan et al., 2019; Pastink et al., 2009). Yet, this study is the first metabolic modelling study that models the different LAB composing a microbial consortium using genome‐scale dynamic metabolic modelling approach. For this purpose, *L. lactis* subsp. *cremoris, L. lactis* subsp. *lactis, Leu. mesenteroides* and *S. thermophilus*, the LAB commonly used in cheese starter cultures, were grown in pure and co‐cultures in chemically defined medium under anaerobic conditions. pH was not controlled in the experiments to mimic cheese fermentation by starter cultures where pH is usually allowed to follow its natural course (Bachmann et al., 2009; Cogan et al., 2007). Co‐cultures comprised of *L. lactis* and *Leu. mesenteroides* strains represent mesophilic cheese starter cultures, while co‐cultures comprised of *L. lactis* and *S. thermophilus* strains represent thermophilic cheese starter cultures. The dynamic metabolic modelling approach implemented here combine both traditional dynamic kinetic modelling and genome‐scale metabolic modelling approaches (Henson & Hanly, 2014). Pure cultures were simulated by dynamic flux balance analysis (dFBA; Mahadevan et al., 2002), while co‐cultures were simulated by the dynamic multi‐species metabolic modelling approach (Hanemaaijer et al., 2017; Zhuang et al., 2011, 2012). As undissociated lactic acid is the main inhibitory component in lactic acid fermentations (Bouguettoucha et al., 2011), substrate uptake kinetics of the dynamic metabolic models was defined with an empirical equation as a function of undissociated lactic acid concentration. The strain‐specific parameters of the substrate uptake kinetics were estimated using pure culture experiments and they were subsequently used to model co‐cultures. The co‐culture models estimated the co‐culture level metabolic capacities and the fermentation dynamics behind the microbial composition of the co‐cultures. Taking advantage of the genome‐scale metabolic modelling, we also used the co‐culture models to elucidate further information on the co‐culture fermentations, which could not be obtained by the experimental methods used, such as individual metabolic exchange flux profiles of the involved organisms and the potential metabolic interactions between LAB in the co‐cultures.

## 2 MATERIALS AND METHODS

### 2.1 Organism and fermentation conditions

LAB used in this study were *L. lactis* subsp. *cremoris* MG1363, *L. lactis* subsp. *lactis* IL1403, *S. thermophilus* LMG 18311 and *L. mesenteroides* subsp. *cremoris* ATCC 19254. Chemically defined medium (CDM) described by (Otto et al., 1983) and modified by (Poolman & Konings, 1988) was used for the preparation of the inoculum and for the fermentations (for the complete component list, see Supporting Information File‐1, Table S1), and the CDM was filter‐sterilized with 0.22 µm filters. Fermentation experiments were carried out under anaerobic conditions in a 1‐L stirred tank bioreactor (Biostat Q, B. Braun Biotech International) with a working volume of 0.6 L at constant temperature and without pH control (initial pH 6.8). The two‐species co‐culture of *L. lactis* subsp. *cremoris* and *Leu. mesenteroides*, and the three‐species co‐culture of *L. lactis* subsp. *cremoris*, *L. lactis* subsp. *lactis* and *Leu. mesenteroides* were assumed to represent mesophilic cheese starter cultures, while the two‐species co‐culture of *L. lactis* subsp. *cremoris* and *S. thermophilus*, and the three‐species co‐culture of *L. lactis* subsp. *cremoris*, *L. lactis* subsp. *lactis* and *S. thermophilus* were assumed to represent thermophilic cheese starter cultures. Pure cultures of *L. lactis, Leu. mesenteroides*, and *S. thermophilus* strains were fermented at 30°C, 30°C and 37°C, respectively, while mesophilic and thermophilic co‐cultures were grown at 30°C and 33°C, respectively. Fermentation medium was flushed with filter‐sterilized N_2_ until dissolved oxygen dropped to zero before inoculation, and there was no gas supply after inoculation. Maintenance of the anaerobic conditions were assumed with slow mixing (50 rpm). For both pure and co‐cultures, the bioreactor was inoculated with 2% (vol/vol) inoculum culture grown till late exponential phase. Initial biomass compositions of the co‐cultures, based on optical density (OD) measurements, were 1:1 (OD:OD) and 1:1:1 (OD:OD:OD) for two and three‐species co‐cultures respectively. For each different batch experiment (pure and co‐cultures), two independent culture replicates were run.

### 2.2 Analytical techniques

Biomass concentration was determined using OD measurements of fermentation culture at 600 nm, which was then correlated with biomass dry weight (gDW). Culture samples were centrifuged at 10,000*g* for 10 min, and cell‐free supernatant was used for glucose, organic acids and amino acids analyses. Biomass samples of co‐cultures were immediately stored at −20°C until the analysis of relative microbial abundance analysis. Glucose concentration was determined by reducing sugar analysis using DNS method (Miller, 1959). Organic acids and amino acids concentrations were determined by high‐performance liquid chromatography as described previously (Özcan et al., 2019). CO_2_ production profiles of *L. lactis* and *S. thermophilus* strains showing homolactic fermentation patterns were assumed to be negligibly small compared to the total carbon outflow under anaerobic conditions as also stated in the literature (Jensen et al., 2001). Molar concentration of ethanol and CO_2_ produced by *Leu. mesenteroides* were estimated based on the glucose and citrate consumption rates as described by our previous study (Özcan et al., 2019).

### 2.3 Estimation of the relative microbial abundance in co‐cultures

Quantitative‐PCR (qPCR) method was employed for quantifying relative microbial abundance ratios of different bacterial strains during co‐culturing. Total cell dry weight concentrations of the co‐cultures were multiplied by the relative microbial abundance ratios to estimate the individual biomass concentrations. DNA extraction was done using peqGOLD Bacterial DNA Kit (Peqlab, VWR), according to manufacturer's protocol, from 3 ml of culture. The primers used for the qPCR procedure (Table 1) are specific to target genomes and iTaq Universal SYBR Green Supermix (Bio‐Rad). The following PCR protocol was used for all samples: initial denaturation at 95°C for 5 min, 40 cycles of 95°C for 15 s, 62°C for 30 s and a melting curve analysis with 0.5°C increments/5 s from 65°C to 95°C using CFX96 Touch Real‐Time PCR Detection System (Bio‐Rad).

**Table 1:** 16S rRNA specific qPCR primers

	Forward primer sequences	Reverse primer sequences	PCR product size	References
*L. lactis* subsp*. cremoris* MG1363	GTGCTTGCACCAATTTGAA	GGGATCATCTTTGAGTGAT	163	Pu et al. (2002)
*L. lactis subsp*. lactis IL1403	GTACTTGTACCGACTGGAT	GGGATCATCTTTGAGTGAT	163	Pu et al. (2002)
*S. thermophilus* LMG 18311	CGGGTGAGTAACGCGTAGGT	CGCCTAGGTGAGCCATTACC	177	This study
*Leu. mesenteroides* ATCC 19254	CCGCATCTTCACGGGTATTT	AGTTTCGGCGAAGGTACGAA	173	This study

### 2.4 Genome‐scale metabolic models (GSMMs) used in this study

The genome‐scale metabolic model (GSMM) of *Leu. mesenteroides* ATCC 19254 (Özcan et al., 2019), *L. lactis* subsp. *cremoris* MG1363 (Flahaut et al., 2013) and the revised version of *S. thermophilus* LMG 18311 (Pastink et al., 2009), which were the same strains as used in experiments, were used in this study. The GSMM of *L. lactis* subsp. *cremoris* MG1363 (Flahaut et al., 2013) was used to simulate the experimental data of both *L. lactis* subsp. *cremoris* and *L. lactis* subsp. *lactis*. In addition to the use of strain‐specific parameters, amino acid auxotrophy of *L. lactis* subsp. *lactis* was also considered for the simulation of this strain. The exchange reactions of arginine, glutamine, histidine, isoleucine, leucine, methionine and valine were constrained in such a way that the model can only consume these amino acids for the simulation of *L. lactis* subsp. *lactis*, because *L. lactis* subsp. *lactis* IL1403 is known to be unable to synthesize these amino acids (Aller et al., 2014; Cocaign‐Bousquet et al., 1995; van Niel & Hahn‐Hägerdal, 1999). The GSMM of *S. thermophilus* LMG 18311 (Pastink et al., 2009) was revised via the following steps: (i) the draft GSMM of *S. thermophilus* LMG 18311 was reconstructed using genome sequence of *S. thermsophilus* LMG 18311 (Bolotin et al., 2004; GenBank accession number GCA_000011825.1) by MetaDraft (B.G. Olivier 2018. [Online], https://systemsbioinformatics.github.io/metadraft) (ii) the new reaction set was compared with that of the original model and the reactions only available in the new draft model was added to the original model to get the revised GSMM of *S. thermophilus* LMG 18311. The revised GSMM of *S. thermophilus* LMG 18311 containing 829 reactions between 886 metabolites governed by 429 genes is available in SBML format in Supporting Information File‐3. Non‐growth associated ATP maintenance rates used in the GSMMs were obtained from original model studies (Flahaut et al., 2013; Oliveira et al., 2005; Özcan et al., 2019; Pastink et al., 2009). For the simulation of the cultures towards the end of the batch, the low glucose uptake rate might not support the original ATP maintenance rate (*m*_ATP_) constraints, which results in an infeasible solution. In such cases, due to modelling purposes, we gradually decreased the *m*_ATP_ value by 0.1 mmol/gDW/h until a feasible solution was obtained. Such a decrease in *m*_ATP_ was also recently demonstrated in a study, where *L. lactis* and *Leu. mesenteroides* were grown in a retentostat reactor as a co‐culture, and *m*_ATP_ values of both species decreased at low growth rates compared to high growth rates (van Mastrigt et al., 2019).

### 2.5 The dynamic metabolic modelling of pure and co‐cultures

**Figure 1:**
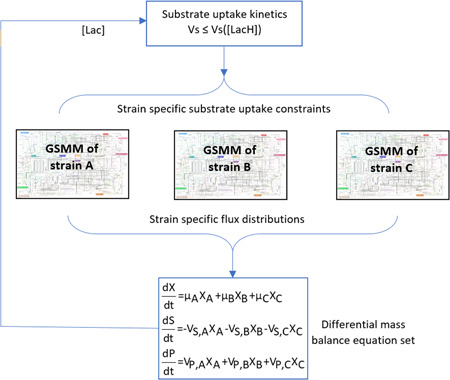
Dynamic co‐culture metabolic modelling approach at genome scale. The system in this example was defined for a three‐species co‐culture. One of the outputs of the solution of the set of differential mass balance equations is co‐culture level lactic acid concentration [*Lac*], which is subsequently used in substrate uptake kinetics, and the dynamic strain‐specific substrate uptake rates constrain the individual GSMMs. *X*, *S* and *P* are biomass, substrate and product concentrations; µ, *V*_S_ and *V*_P_ are growth, substrate uptake and product production rates, respectively. [Color figure can be viewed at wileyonlinelibrary.com]

Concentration profiles of biomass and extracellular metabolites of batch cultures were simulated by dynamic metabolic modelling approaches. Static optimization‐based dynamic flux balance analysis (dFBA) approach (Mahadevan et al., 2002) was applied for the pure culture of *L. lactis* subsp. *cremoris*, *L. lactis* subsp. *lactis*, *S. thermophilus* and *Leu. mesenteroides*, while dynamic co‐culture metabolic modelling approach (Hanemaaijer et al., 2017; Zhuang et al., 2011, 2012) which is a dFBA approach adapted for multi‐species systems, was applied for the co‐cultures (Figure 1). In dynamic models, differential mass balances were written for the following extracellular metabolites: the metabolites experimentally measured (glucose, organic acids and amino acids), other metabolites in the fermentation medium (vitamins and nucleic acids) and the metabolites that are known to be produced (flavour compounds).

Undissociated form of lactic acid inhibited growth and the model growth is limited by substrate uptake. To mimic that effect, substrate uptake kinetics of the dynamic models was defined with an empirical equation, which was a function of undissociated lactic acid concentration:

(1) $$V_{i}\leq -V_{\max,i}\text{exp}(-K_{\mathrm{LacH},i}[\mathrm{LacH}])-V_{\min,i}$$

where *i* denotes the index for glucose or amino acids, *V* is substrate uptake rate, *V*_max_, *V*_min_ and *K*_LacH_ are the parameters that denote maximum uptake rate, minimum uptake rate and undissociated lactic acid constant, respectively. In GSMMs, negative values of exchange reaction rates denote consumption, and positive values of the exchange reaction rates denote production for the corresponding compound. Substrate uptake kinetics constrained only lower bound of the substrate utilization rates.

According to the Henderson–Hasselbalch equation (Bouguettoucha et al., 2011), the relationship between undissociated and total lactic acid can be written as:

(2) $$[\mathrm{LacH}]=\frac{\left[\mathrm{Lac}\right]}{1+\text{exp}(\mathrm{pH}‐pK_{a})}$$

where [LacH], [Lac] and p*K*_a_ are undissociated lactic acid concentration, total lactic acid concentration and logarithmic acid dissociation constant, respectively. Assuming pH to be linearly correlated with total lactic acid concentration, [LacH] term in Equation (2) can be written as:

(3) $$[\mathrm{LacH}]=\frac{\left[\mathrm{Lac}\right]}{1+\text{exp}(C_{1}[\mathrm{Lac}]+C_{2})}$$

The constants, *C*_1_ and *C*_2_ in Equation (3) were estimated by the non‐linear regression of batch‐specific experimental [LacH] and [Lac] values in mmol/L. Finally, combining Equations (1) and (3) substrate uptake kinetics used in dynamic models leads to:

(4) $$V\leq -V_{\max}\text{exp}(-K_{\mathrm{LacH}}\;\frac{\left[\mathrm{Lac}\right]}{1+\text{exp}(C_{1}[\mathrm{Lac}]+C_{2})})-V_{\min}$$

Metabolic flux distribution in the dynamic models was calculated by two sequential optimizations. The first one is a linear programming (LP) problem (i.e., flux balance analysis, FBA; Orth et al., 2010) which maximizes the growth rate by constraining the models with the carbon source and amino acid utilization rates, and the secondary optimization is a quadratic programming (QP) problem that minimizes the total sum of absolute fluxes. QP applied in the flux analyses as a secondary optimization after LP is based on the principle of minimal use of enzyme resources to achieve the primary objective, and it also helps to avoid the alternate optima problem (Lewis et al., 2010; Tarlak et al., 2014). Metabolic flux analyses were performed using COBRA Toolbox (Schellenberger et al., 2011) in MATLAB environment, with Gurobi6 (http://www.gurobi.com) as the optimization solver. The ordinary differential equation (ODE) sets in dynamic models were solved by ode45, which is a MATLAB function based on the Runge‐Kutta numerical method. Finally, experimentally obtained initial biomass and metabolite concentrations were used as initial conditions for the solution of ODE sets.

The parameters of the substrate uptake kinetics in Equation (1) were dynamically estimated by MEIGO optimization tool (Egea et al., 2014). The strain‐specific kinetic parameters were estimated using the pure culture experiments, and they were used both in the pure and co‐culture models.

## 3 RESULTS

### 3.1 Dynamic pure cultures metabolic models

The pure cultures of *L. lactis* subsp. *cremoris*, *L. lactis* subsp. *lactis*, *S. thermophilus* and *Leu. mesenteroides* were fermented until stationary phase under anaerobic conditions. The pure cultures of *L. lactis* and *S. thermophilus* species showed homolactic fermentation in which the main fermentation product was lactic acid, while *Leu. mesenteroides*, which is an obligate heterolactic lactic acid bacterium (Özcan et al., 2019), produced CO_2_ and ethanol in addition to lactic acid. The difference between homolactic and heterolactic fermentation could also be manifested by the yield of lactic acid produced per glucose consumed, which was lowest for *Leu. mesenteroides* among all pure culture batches (Supporting Information File‐1, Table S2). To mimic the cheese fermentation, pH was not controlled for all batches, and the microbial growth was inhibited by undissociated lactic acid which is increasingly formed by low pH. Therefore, glucose and amino acids were not consumed completely in all batches. On the other hand, during the stationary phase at such low pH levels, glucose consumption and lactic acid production slightly continued. The yield of lactic acid produced per glucose consumed was almost constant during all batches (Supporting Information File‐1, Figure S1).

Biomass and extracellular metabolites concentration profiles of the pure cultures of *L. lactis*, *S. thermophilus* and *Leu. mesenteroides* strains were then simulated by dFBA. The strain specific parameters of the substrate uptake kinetics used in the dynamic models were estimated by dynamic parameter estimation (Supporting Information File‐2). The lower bound of the substrate uptake rates (which are defined in GSMMs as negative fluxes) was fixed to the values obtained through substrate uptake kinetics, while the upper bound of the substrate uptake rates was free to let the model consume less, or even produce the corresponding substrate. In addition to the glucose and amino acids uptake rate constraints, the experimental glucose to lactic acid yield was also used as a constraint by fixing the ratio between glucose consumption and lactic acid production rates in GSMMs of *L. lactis* and *S. thermophilus*, which assured *in‐silico* homolactic fermentation as observed experimentally. Glucose to lactic acid yield as a model constraint was implemented using the *addRatioReaction* function of COBRA Toolbox (Schellenberger et al., 2011). Without such a lactic acid yield constraint, *L. lactis* and *S. thermophilus* models showed mixed acid fermentation, which produced acetic acid, formic acid and/or ethanol instead of lactic acid, as previously reported in the GSMM studies of *Lactobacillus plantarum* (Teusink et al., 2006) and *L. lactis* (Flahaut et al., 2013; Oliveira et al., 2005). The reason behind the mixed acid fermentation preference of the models with biomass optimization is extra ATP gain with acetic acid production, and re‐oxidization of NADH through formic acid and ethanol production in mixed acid fermentation. GSMM of *Leu. mesenteroides* did not need the lactic acid yield constraint as the organism is an obligate heterolactic fermentative lactic acid bacterium that uses the phosphoketolase pathway and produces lactic acid and ethanol in anaerobic fermentation for ATP production and reoxidation of NADH, respectively (Özcan et al., 2019).

**Figure 2:**
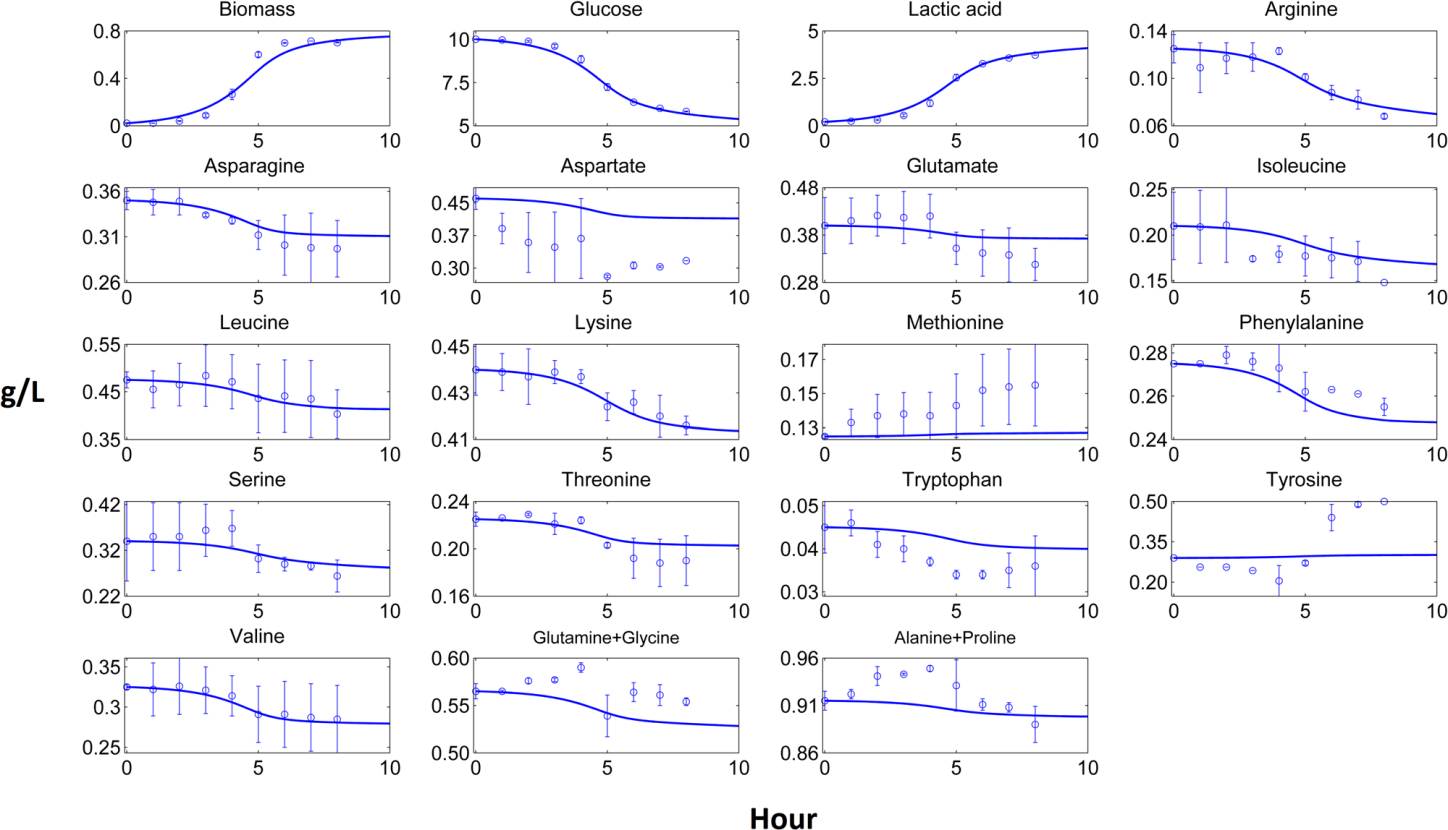
Computational and experimental batch culture concentration profiles of *Lactococcus lactis* subsp. *cremoris*. Solid lines denote model results simulated by dynamic flux balance analysis (dFBA), while points and bars denote average and range of two biological replicates, respectively [Color figure can be viewed at wileyonlinelibrary.com]

**Figure 3:**
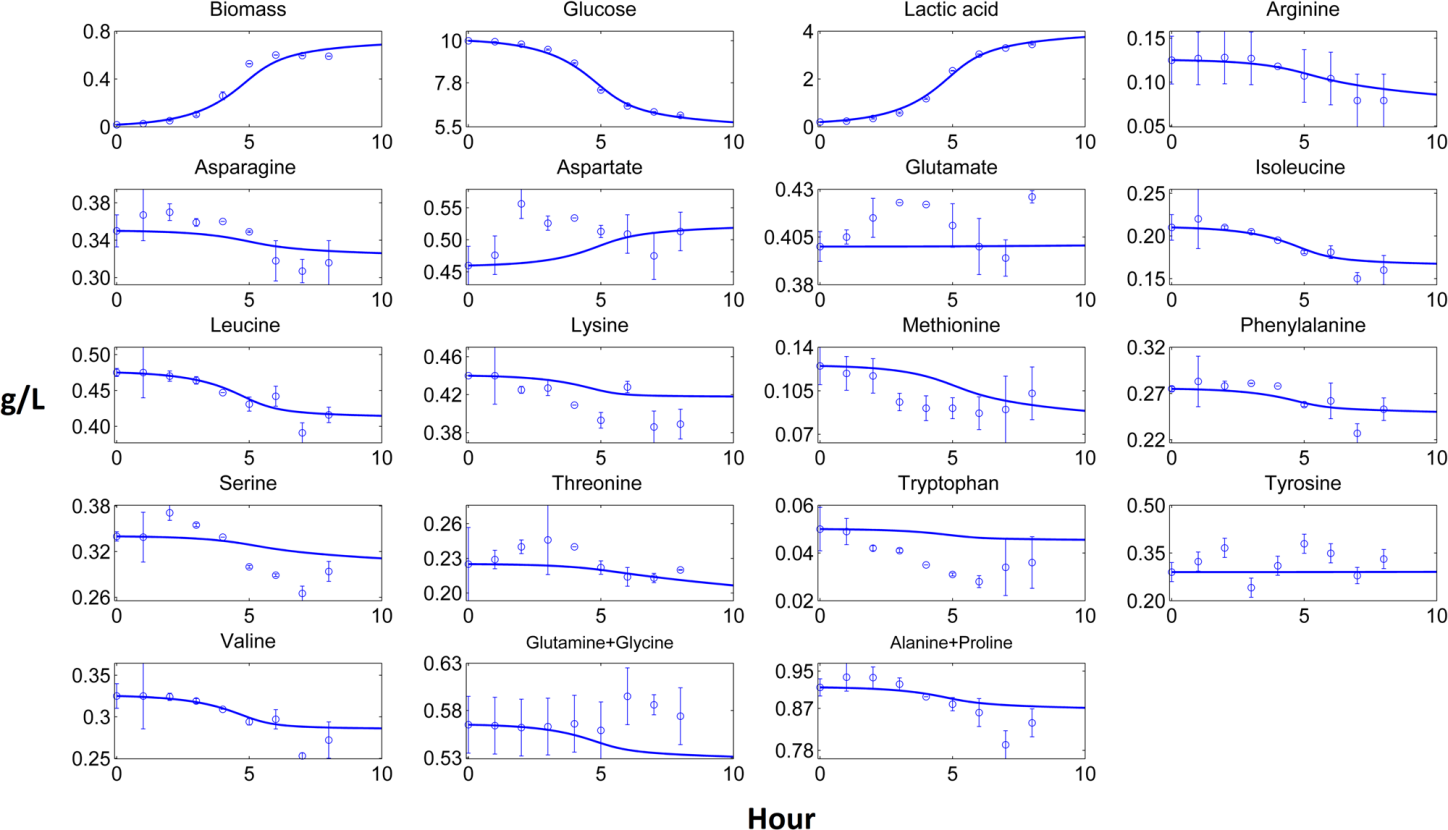
Computational and experimental batch culture concentration profiles of *Lactococcuslactis* subsp. *lactis*. Solid lines denote model results simulated by dynamic flux balance analysis (dFBA), while points and bars denote average and range of two biological replicates, respectively [Color figure can be viewed at wileyonlinelibrary.com]

**Figure 4:**
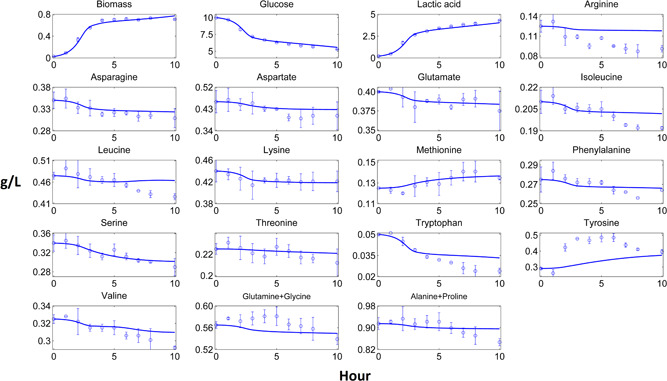
Computational and experimental batch culture concentration profiles of *Streptococcus thermophilus*. Solid lines denote model results simulated by dynamic flux balance analysis (dFBA), while points and bars denote average and range of two biological replicates, respectively [Color figure can be viewed at wileyonlinelibrary.com]

**Figure 5:**
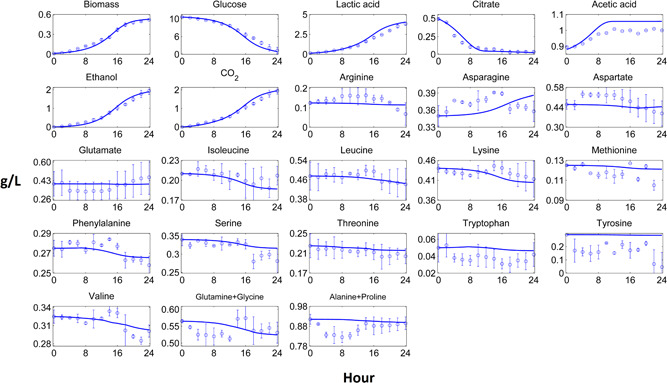
Computational and experimental batch culture concentration profiles of *Leu. mesenteroides*. Solid lines denote model results simulated by dynamic flux balance analysis (dFBA), while points and bars denote average and range of two biological replicates, respectively [Color figure can be viewed at wileyonlinelibrary.com]

Dynamic pure culture model results of biomass, carbon source, fermentation products and most of the amino acid profiles fitted closely to the experimental data (Figures 2, 3, 4, 5). These results pointed out that the use of the substrate uptake kinetics as a function of undissociated lactic acid in the dynamic metabolic modelling was a suitable approach to define the dynamics of the cheese starter cultures. Experimental amino acid profiles of the pure cultures were mostly coupled with biomass profiles (Figures 2, 3, 4, 5). The chromatography profiles of glutamine/glycine and alanine/proline pairs overlapped so that their concentration profiles were given as the sum of the corresponding pair. For some amino acids such as aspartate in the *L. lactis* subsp. *cremoris* culture, the models underestimated the experimental profiles. The underestimated *in‐silico* amino acid profiles showed that the models used less amino acids than those observed in the experiments. Excess amino acids consumption that could not be predicted by the models might be due to their use in other metabolic pathways not considered by these models. Methionine and tyrosine were experimentally produced in *L. lactis* subsp. *cremoris* and *S. thermophilus* batches. These amino acids were underestimated by the *L. lactis* model as the production of these amino acids dramatically decreased the *in‐silico* growth. On the other hand, aspartate and asparagine were experimentally produced by *L. lactis* subsp. *lactis* and *Leu. mesenteroides*, respectively, and the models of these strains could estimate the productions.

Citrate was not consumed significantly in *L. lactis* and S*. thermophilus* strains (results not shown), while *Leu. mesenteroides*, which is known as a citrate consumer (Smid & Kleerebezem, 2014), consumed all citrate before the stationary phase. The source of the acetic acid produced in *Leu. mesenteroides* was the citrate consumed through citrate utilization pathway (Özcan et al., 2019). Heterolactic fermentation products, ethanol and CO_2_, produced through phosphoketolase pathway were also simulated by the dynamic model of *Leu. mesenteroides* (Figure 5).

### 3.2 Dynamic co‐culture metabolic models

We simulated mesophilic and thermophilic co‐cultures by a previously described dynamic co‐culture metabolic modelling approach (Hanemaaijer et al., 2017; Zhuang et al., 2011, 2012). We used the strain‐specific parameters estimated using the pure culture experiments (Supporting Information File‐2, Table S1), and only estimated the co‐culture specific parameters, *C*_1_ and *C*_2_ (Supporting Information File‐2, Table S2) that describe the co‐culture specific pH profiles.

**Figure 6:**
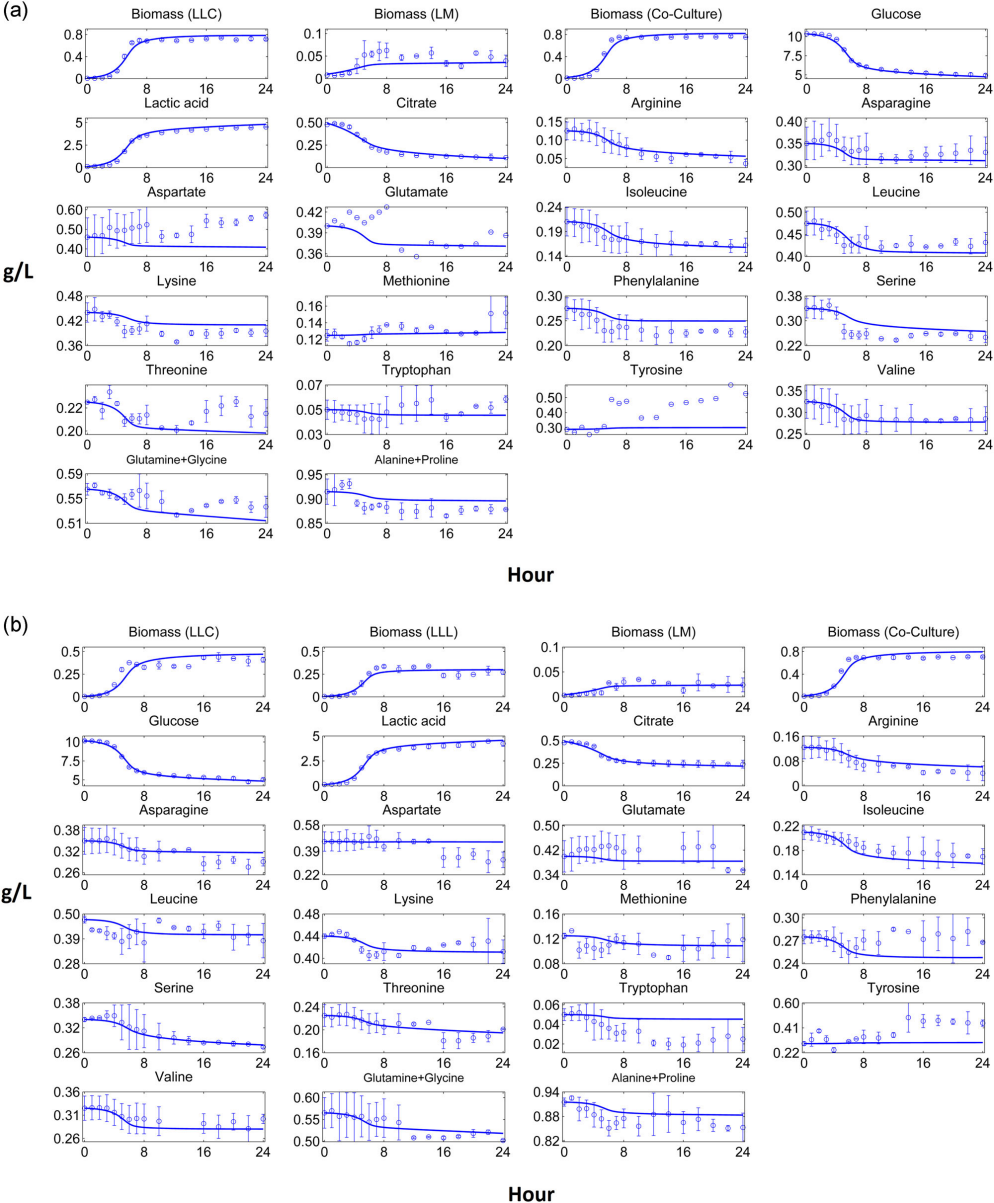
Computational and experimental mesophilic co‐culture profiles. (a) Two‐species mesophilic co‐culture comprised of *L. lactis* subsp. *cremoris* (LLC) and *Leu. mesenteroides* (LM). (b) Three‐species mesophilic co‐culture comprised of LLC, *L. lactis* subsp. *lactis* (LLL) and LM. Solid lines denote the co‐culture model results, while points and bars denote average and range of two biological replicates, respectively [Color figure can be viewed at wileyonlinelibrary.com]

Mesophilic co‐culture models fitted closely to the experimental data. The mesophilic co‐culture models and experiments showed the domination of *L. lactis* species over *Leu. mesenteroides*. The contribution of *Leu. mesenteroides* to the final biomass in two and three species co‐cultures were around 6% and 3.5%, respectively (Figure 6). This result is consistent with the previous reports (Erkus et al., 2013; van Mastrigt et al., 2019) stating that the final biomass ratio of *Leu. mesenteroides* in long term (days) is around 1% in the mesophilic cheese starter cultures comprised of *L. lactis* and *Leu. mesenteroides* strains.

The suppression of *Leu. mesenteroides* in the co‐cultures could be explained by the rapid acidification of the medium by *L. lactis* strains. In other words, lactic acid pool mostly produced by *L. lactis* strains decreased the uptake rates of *Leu. mesenteroides* according to the substrate uptake kinetics, which made *Leu. mesenteroides* disadvantageous in the competition for sugar and amino acid source in co‐cultures. Another reason for the suppression of *Leu. mesenteroides* could be the ATP yield of *Leu. mesenteroides* per mole of glucose consumed. *Leu. mesenteroides* is an obligate heterolactic lactic acid bacterium, and ATP yield of the obligate heterolactic fermentation is lower than homolactic fermentation as observed in *L. lactis* (Ganzle, 2015). On the other hand, unlike the pH‐controlled co‐culture of *L. lactis* and *Leu. mesenteroides* reported in literature (van Mastrigt et al., 2019), the ATP yield was a minor explanation for the growth suppression of *Leu. mesenteroides* in our study, as the effect of rapid acidification was dominant. Final biomass concentrations of *Leu. mesenteroides* in mesophilic co‐cultures were 10‐fold less than the ones in pure culture, which was also reported in a study investigating *L. lactis* and *Leu. mesenteroides* strains in pure and co‐cultures in reconstituted skim milk (Bellengier et al., 1997). Furthermore, final biomass concentration of *L. lactis* subsp. *lactis* was lower than *L. lactis* subsp. *cremoris* in the three‐species mesophilic co‐culture, as observed in pure cultures.

**Figure 7:**
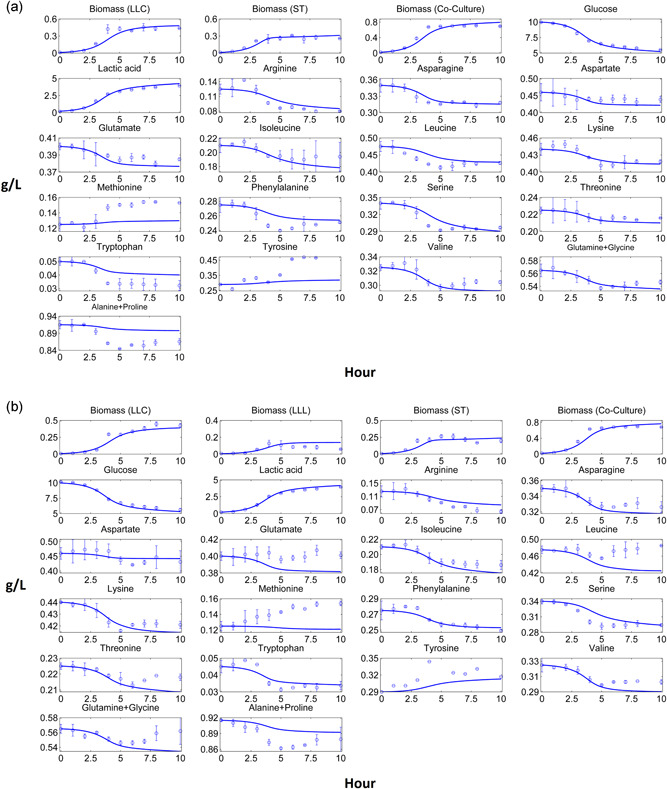
Computational and experimental thermophilic co‐culture profiles. (a) Two‐species thermophilic co‐culture comprised of *Lactococcus lactis* subsp. *cremoris* (LLC) and *S. thermophilus* (ST). (b) Three‐species thermophilic co‐culture comprised of LLC, *L. lactis* subsp. *lactis* (LLL) and ST. Solid lines denote the co‐culture model results, while points and bars denote average and range of two biological replicates, respectively [Color figure can be viewed at wileyonlinelibrary.com]

Similar to the mesophilic co‐cultures, *L. lactis* subsp. *cremoris* dominated the thermophilic co‐cultures experimentally, but the preliminary analyses of thermophilic co‐culture models showed the opposite, with *S. thermophilus* dominating the co‐cultures in‐silico (results not shown). In addition to a possible interaction between *S. thermophilus* and *L. lactis*, which the co‐culture models might miss, this unexpected result could be explained by the difference in the fermentation temperature of *L. lactis* and *S. thermophilic* in pure and thermophilic co‐cultures, which were 30°C, 37°C and 33°C respectively (see methods). In terms of the growth performance based on the experimental growth rates with respect to pH values (Supporting Information File‐1, Figure S2), *L. lactis* showed  higher and *S. thermophilus* showed a lower growth performance in thermophilic cocultures compared to their pure cultures at above the pH values causing the inhibitory effect (i.e., ~pH ≤ 5). This result is consistent with the reported effect of different temperatures on growth rate for several LAB including *L. lactis* and *S. thermophilus* at optimal pH values of related species (Adamberg et al., 2003). Because of the temperature difference, the growth rate profile of *L. lactis* in the co‐culture increased around 20%, while the growth rate profile of *S. thermophilus* in the co‐culture decreased around 20%, compared to their pure cultures. Assuming the substrate uptake rates are coupled with growth rate, all substrate uptake rates of *L. lactis* and *S. thermophilus* were then multiplied by 1.2 and 0.8, respectively, in coculture simulations to consider the effect of temperature difference. After multiplying all the substrate uptake rates by the correction coefficients, the domination of *L. lactis* subsp. *cremoris* in thermophilic co‐cultures could be simulated (Figure 7). Two‐species thermophilic co‐culture model fitted closely to the experimental concentration profiles of individual biomass and coculture level extracellular compounds (Figure 7a). Unlike mesophilic co‐cultures, acidification did not affect the biomass composition of the thermophilic co‐culture significantly, as both *L. lactis* and *S. thermophilus* species showed similar homolactic fermentation patterns. The final biomass composition of two‐species thermophilic co‐culture was around 1:0.6 (*L. lactis* subsp*. cremoris*:*S. thermophilus*). Although individual biomass abundance ratio of *L. lactis* subsp. *cremoris* decreased in the early phase of the batch, its abundance increased afterwards, and this experimental result was also predicted by the model (Supporting Information File‐1, Fig. S3).

Although the three‐species thermophilic co‐culture model predicted the final individual biomass‐based compositions of the organisms as *L. lactis* subsp. *cremoris* being the most and *L. lactis* subsp. *lactis* being the least abundant species, the model could not predict the individual biomass profiles of *L. lactis* strains precisely (Figure 7b). Experimentally obtained individual biomass profiles in the co‐cultures were not as smooth as those observed in pure cultures. We observed some fluctuations on the individual biomass profiles of the co‐culture especially at stationary phase, which could be due to the estimation method of the relative microbial abundance (see methods). These small and instantaneous increase/decrease at the individual biomass profiles of all co‐cultures at stationary phases were assumed acceptable as they showed the general biomass dynamics of the co‐culture. Experiments showed that glutamate, leucine and threonine were produced at the late phase of the three‐species thermophilic co‐culture, but the model could not simulate these productions due to the objective function in the models, which forced the consumption of these amino acids to ensure the maximum *in‐silico* growth rate.

### 3.3 In‐silico production profiles of flavour compounds in co‐cultures

**Figure 8:**
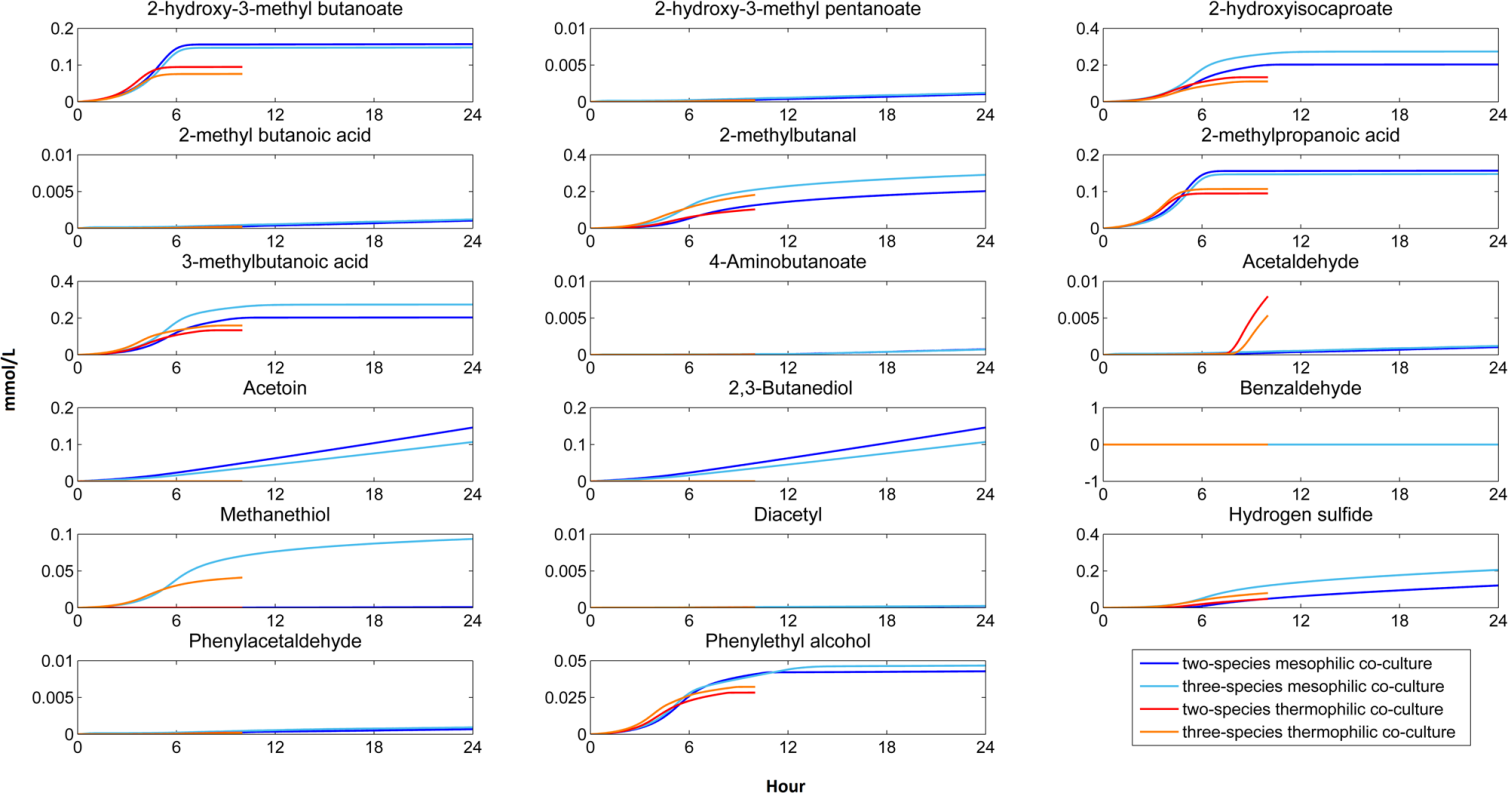
In‐silico flavour compound production profiles of two and three‐species mesophilic and thermophilic co‐cultures [Color figure can be viewed at wileyonlinelibrary.com]

In‐silico production profiles of the flavour compounds in the co‐cultures were estimated through the pathways already defined in the GSMMs used (Figure 8). Maximum production rates of the flavour compounds calculated by FVA (Mahadevan & Schilling, 2003) were used in the dynamic models to estimate the maximum flavour compounds production potential of the co‐cultures. Flavour metabolites acetoin, diacetyl, 2,3‐butanediol, acetaldehyde and benzaldehyde are produced through pyruvate metabolism while the rest of the flavour metabolites in Figure 8 are produced through amino acid catabolism. Detailed information for the pathways of flavour metabolites produced by LAB can be found elsewhere (Smid & Kleerebezem, 2014; Smit et al., 2005; Yvon & Rijnen, 2001). Production of acetoin, diacetyl and 2,3‐butanediol by *Leu. mesenteroides* and acetaldehyde production by *S. thermophilus* are well known phenomena in fermentative dairy foods (Bottazzi & Dellaglio, 1967; Hemme & Foucaud‐Scheunemann, 2004), and these metabolites are the only flavour compounds defined in the corresponding GSMMs. On the other hand, all flavour compounds in Figure 8 are defined in the GSMM of *L. lactis*.

Parallel with the amino acid consumption limitation due to low pH, production of *L. lactis*‐origin flavour metabolites 2‐hydroxy‐3‐methyl‐butanoate, 2‐hydroxyisocaproate, 2‐methylbutanal, 2‐methylpropanoic acid, 3‐methyl‐butanoic acid, methanethiol and phenylethyl alcohol decreased or stopped at stationary phase. The rest of *L. lactis*‐origin flavour metabolites were not produced at significant level (Figure 8). Methanethiol is produced through methionine catabolism (Flahaut et al., 2013), and in‐silico methanethiol production was only observed in three‐species mesophilic and thermophilic co‐cultures, as *L. lactis* subsp. *lactis* in the three‐species co‐cultures was the only species that consumed methionine. *L. lactis*‐origin benzaldehyde and *Leu. mesenteroides*‐origin diacetyl were not produced since oxygen is required to produce these compounds according to the corresponding GSMMs. Production of acetoin and 2,3‐butanediol was only observed in mesophilic co‐cultures, as these compounds are originated from *Leu. mesenteroides*. Although production of *L. lactis*‐origin flavour compounds, produced through amino acid catabolism, was inhibited by low pH, production of *Leu. mesenteroides*‐origin flavour compounds, produced through pyruvate metabolism, continued during the entire batch. In our previous study, flavour formation by *Leu. mesenteroides* metabolic model was observed after the carbon source uptake did not enhance the growth rate anymore, in other words, flavour compound production only occurred under carbon and ATP excess (Özcan et al., 2019). In the current study, both experimentally and computationally, carbon source consumption slightly continued after growth inhibition for all batches, and the growth inhibition of *Leu. mesenteroides* in mesophilic co‐cultures started at the early stage of the batches, and a certain part of the carbon source consumed by *Leu. mesenteroides* was used for the production of flavour compounds. This *in‐silico* result is consistent with the study that showed that acidic conditions promoted the acetoin production by *Leu. mesenteroides* (LevataJovanovic & Sandine, 1996).

### 3.4 Potential metabolic interactions in the co‐cultures

In the previous sections, we used the co‐culture models to estimate the co‐culture level metabolite profiles. However, the co‐culture models can also estimate the contribution of individual organisms on the total consumption/production of a metabolite in co‐culture medium. This is a crucial information about microbial consortia and is not easily obtained by experiments. Moreover, such metabolite consumption/production profiles of the individual organisms can point out the potential metabolic interactions in a co‐culture. For instance, a metabolite produced by an organism and consumed by other(s) in a co‐culture has cross‐feeding potential between the organisms.

**Figure 9:**
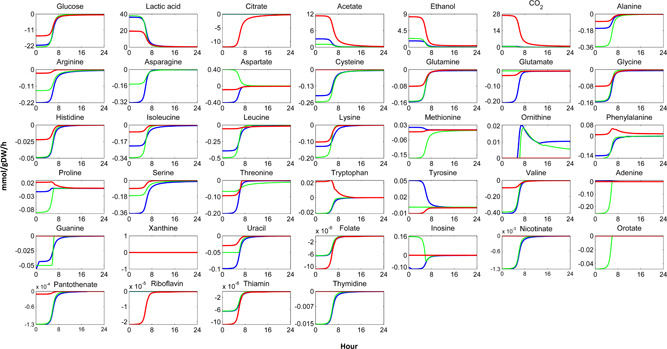
Individual exchange flux profiles profiles of *Lactococcus lactis* subsp. *cremoris* (blue line), *L. lactis* subsp. *lactis* (green line) and *Leu. mesenteroides* (red line) in three‐species mesophilic co‐culture. Negative and positive flux values show consumption and production, respectively [Color figure can be viewed at wileyonlinelibrary.com]

To this end, we used the co‐culture models to estimate the exchange flux profiles of various metabolites of the individual organisms in the co‐cultures. Metabolic exchange flux profiles estimated by the co‐culture models showed that, in the two‐species mesophilic co‐culture, asparagine, methionine, proline and tryptophan were produced by one organism, while the other consumed them (Supporting Information File‐1, Figure S4). In the three‐species mesophilic co‐culture, asparagine, aspartate, methionine, phenylalanine, proline, tryptophan and inosine were the metabolites that one organism produced while the others consumed or vice versa (Figure 9). In the two‐species thermophilic co‐culture, leucine and adenine were estimated by the model as being cross‐fed between *L. lactis* and *S. thermophilus* (Supporting Information File‐1, Figure S5). In the three‐species thermophilic co‐culture, the model estimated that aspartate was produced and methionine was consumed by *L. lactis* subsp. *lactis* and leucine was slightly produced by *S. thermophilus* at stationary phase, which was a different metabolic behaviour than the other co‐culture members (Supporting Information File‐1, Figure S6).

## 4 DISCUSSION

This study showed that monoculture level properties could be used to understand the co‐culture level properties for the LAB consortia. We estimated the strain‐specific kinetic parameters using the monocultures experiments and used these parameters in the co‐culture models. The dynamic co‐culture models then accurately predicted the experimental results such as dynamic biomass compositions and the concentration profiles of glucose and lactic acid. Conventional kinetics expressions based on an enzyme‐substrate relationship such as the Michaelis–Menten kinetics could not explain our system (results not shown), as the substrate itself was not a rate limiting compound in our system where none of the substrates were totally consumed due to pH inhibition. Hence, we defined the substrate uptake kinetics of the dynamic models with an empirical equation as a function of undissociated lactic acid concentration, and it was a suitable kinetic expression for the fermentations without pH control. Due to the mathematical nature of the substrate uptake kinetics (see methods), the models start fermentation with maximum substrate uptake rate values in the early stage of the batches, where undissociated lactic acid concentrations were below the rate limiting level. This could have been a disadvantage for fermentations with long lag phases, but in our case the lag phase period of the batches were short enough to be ignored, and fits were good.

In addition to the metabolite profiles that were already obtained by experiments, the co‐culture models can estimate the profile of more metabolites that could not be obtained by the experimental methods used, such as nucleic acids, vitamins and flavour compounds. Since one of the main functions of the starter cultures is flavour compound production (Smid & Kleerebezem, 2014; Smit et al., 2005), we used the co‐culture models to estimate the flavour compound production profiles of mesophilic and thermophilic co‐cultures through the pathways already defined in the GSMMs used. Another modelling output that could not obtained by experimental methods used is the potential metabolic interaction between the LAB in the co‐cultures. We obtained co‐culture level extracellular metabolite profiles experimentally, and the co‐culture models estimated the contribution of individual organisms on these profiles through the metabolic exchange flux profiles of the individual organisms in the co‐cultures. This also revealed the potential metabolic interactions between the LAB in the co‐cultures. Co‐culture models estimated the amino acids as the major exchanged metabolites between organisms in the related co‐cultures. Amino acid exchange between organisms is one of the important interactions in dairy cultures, and it mostly occurs through the proteolysis of the casein by proteolytic strains (Smid & Lacroix, 2013). Unlike the chemically defined medium that we also used for the fermentations, complex media such as milk evolutionarily pave the way for more co‐operational metabolic interactions, which can be benefited from by either individual organisms or entire consortia. In yoghurt culture, non‐proteolytic *S. thermophilus* consumes peptide and amino acid that are released by proteolytic *Lactobacillus bulgaricus*, in turn *S. thermophilus* supplies some growth stimulating factors to *L. bulgaricus* such as formic acid and folic acid (Sieuwerts et al., 2008). In yeast‐LAB consortia such as kefir culture, yeast can benefit from galactose which is secreted by LAB as a result of lactose catabolism, in turn LAB benefits from amino acids secreted by yeast (Ponomarova et al., 2017).

Biomass and lactic acid yields of the co‐cultures in this study were in between the yields of pure cultures that constitute the related co‐cultures (Supporting Information File‐1, Table S2). This result showed that the metabolic interaction in the co‐cultures did not create an advantage in terms of the yields, which is contrary to the ones observed in other dairy cultures such as yoghurt and kefir. This could be due to the lack of common evolutionary history of the strains used and the chemically defined medium used, which contains all compounds required for cell growth. However, the estimation of potential metabolic interactions is still important for such experimental set‐ups to design better starter cultures. It is noteworthy to mention that the potential metabolic interactions estimated by the co‐culture models are study‐specific results, and they should not be generalized for all cheese starter cultures since amino acid, vitamin and carbon source auxotrophies of LAB to be used in the dairy starter cultures vary across different LAB (Teusink & Molenaar, 2017). On the other hand, the dynamic co‐culture metabolic modelling applied in this study can be used as a promising approach to uncover potential metabolic interactions in co‐cultures having no known metabolic interactions.

## AUTHOR CONTRIBUTIONS

Emrah Özcan, Tunahan Çakır, Ebru Toksoy Öner and Bas Teusink conceived and designed the study. Emrah Özcan performed all computational and experimental studies except qPCR and amino acid analysis. Merve Seven performed qPCR analysis. Burcu Şirin performed amino acid analysis. Emrah Özcan, Emrah Nikerel, Tunahan Çakır, Ebru Toksoy Öner and Bas Teusink discussed the results and wrote the manuscript.

## Supporting information

Supporting information.

Supporting information.

Supporting information.
