# Supplementary material for: Dynamic co‐culture metabolic models reveal the fermentation dynamics, metabolic capacities and interplays of cheese starter cultures
Source: Biotechnol Bioeng. 2020 Sep 28;118(1):223–37. doi: 10.1002/bit.27565 (PMC7971941; doi:10.1002/bit.27565)
Supplement: Supplementary file 1 — Supporting information. [file BIT-118-223-s001.docx]

**Dynamic co-culture metabolic models reveal the fermentation dynamics, metabolic capacities and interplays of cheese starter cultures**

**Emrah Özcan^1,2^, Merve Seven^3^, Burcu Şirin^3^, Tunahan Çakır^4^, Emrah Nikerel^3^, Bas Teusink^1^* and Ebru Toksoy Öner^2^**

^1^ Systems Biology, Amsterdam Institute of Molecular and Life Sciences (AIMMS), VU Amsterdam, Amsterdam, The Netherlands

^2^ IBSB, Department of Bioengineering, Marmara University, Istanbul, Turkey

^3^ Genetics and Bioengineering Department, Yeditepe University, Istanbul, Turkey

^4^ Department of Bioengineering, Gebze Technical University, Gebze, Kocaeli, Turkey

* Corresponding author

**Table S1.** Chemically defined medium (CDM) composition used in this study.

|  | (g/L) |  | (g/L) |
| --- | --- | --- | --- |
| **Main Ingredients** |  | **DNA precursor mix** |  |
| Glucose | 10 | Adenine | 0.01 |
| K_2_HPO_4_ | 2.5 | Guanine | 0.01 |
| KH_2_PO_4_ | 3 | Xanthine | 0.01 |
| Na-acetate | 1 | Uracil | 0.01 |
| (NH_4_)_3_-citrate | 0.6 | **Vitamins** |  |
| Ascorbic acid | 0.5 | Pyridoxamine-HCl | 0.005 |
| **Amino acids** |  | D-biotin | 0.01 |
| Alanine | 0.24 | 6,8-thioctic acid | 0.0025 |
| Arginine | 0.125 | Pyridoxine-HCl | 0.002 |
| Asparagine | 0.35 | Nicotinic acid | 0.001 |
| Aspartate | 0.46 | Ca-(D+) pantothenate | 0.001 |
| Cysteine | 0.25 | Riboflavin | 0.001 |
| Glutamate | 0.4 | Thiamin-HCl | 0.001 |
| Glutamine | 0.39 | Vitamin B_12_ | 0.001 |
| Glycine | 0.175 | Na-4-aminobenzoate | 0.01 |
| Histidine | 0.15 | Orotic acid | 0.005 |
| Isoleucine | 0.21 | 2-deoxythymidine | 0.005 |
| Leucine | 0.475 | Inosine | 0.005 |
| Lysine | 0.44 | Folic acid | 0.001 |
| Methionine | 0.125 | **Trace metals** |  |
| Phenylalanine | 0.275 | MgCl_2_ × 6 H_2_O | 0.2 |
| Proline | 0.675 | CaCl_2_ × 2 H_2_O | 0.05 |
| Serine | 0.34 | MnSO_4_ x H_2_O | 0.028 |
| Threonine | 0.225 | FeCl_2_ × 4 H_2_O | 0.005 |
| Tryptophan | 0.05 | ZnSO_4_ × 7 H_2_O | 0.005 |
| Tyrosine | 0.29 | CoCl_2_ × 6 H_2_O | 0.0025 |
| Valine | 0.325 | CuSO_4_ × 5 H_2_O | 0.0001 |

**Table S2.** The fermentation yields of pure and co-cultures.

| Batch | Y_X/S_, Biomass yield (g Biomass/ g Glc) * | Y_P/S_, Lactic acid yield (g Lac/ g Glc) |
| --- | --- | --- |
| *L. lactis* subsp. *cremoris* (LLC) | 0.186±0.001 | 0.841±0.007 |
| *L. lactis* subsp. *lactis* (LLL) | 0.171±0.001 | 0.833±0.000 |
| *S. thermophilus* (ST) | 0.183±0.006 | 0.865±0.010 |
| *Leu. mesenteroides* (LM) | 0.068±0.006 | 0.487±0.037 |
| Two species mesophilic co-culture (LLC-LM) | 0.182±0.005 | 0.805±0.026 |
| Three-species mesophilic co-culture (LLC-LLL-LM) | 0.173±0.006 | 0.802±0.031 |
| Two-species thermophilic co-culture (LLC-ST) | 0.181±0.001 | 0.855±0.011 |
| Three-species thermophilic co-culture (LLC-LLL-ST) | 0.175±0.005 | 0.826±0.031 |

*Since glucose consumption continued after growth inhibition, biomass yields were based on exponential growth phase.


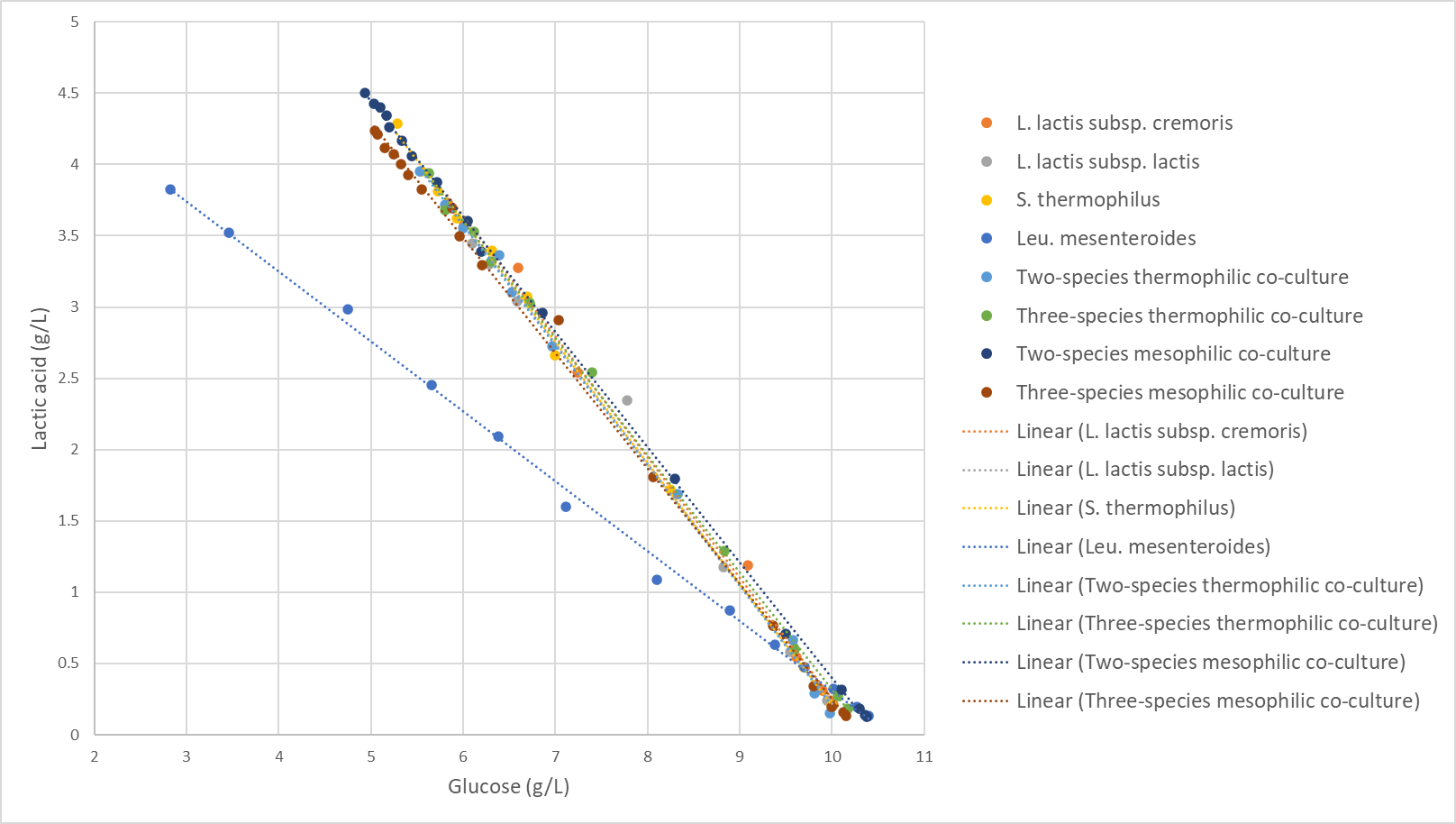


**Figure S1.** Lactic acid produced with respect to glucose consumed.


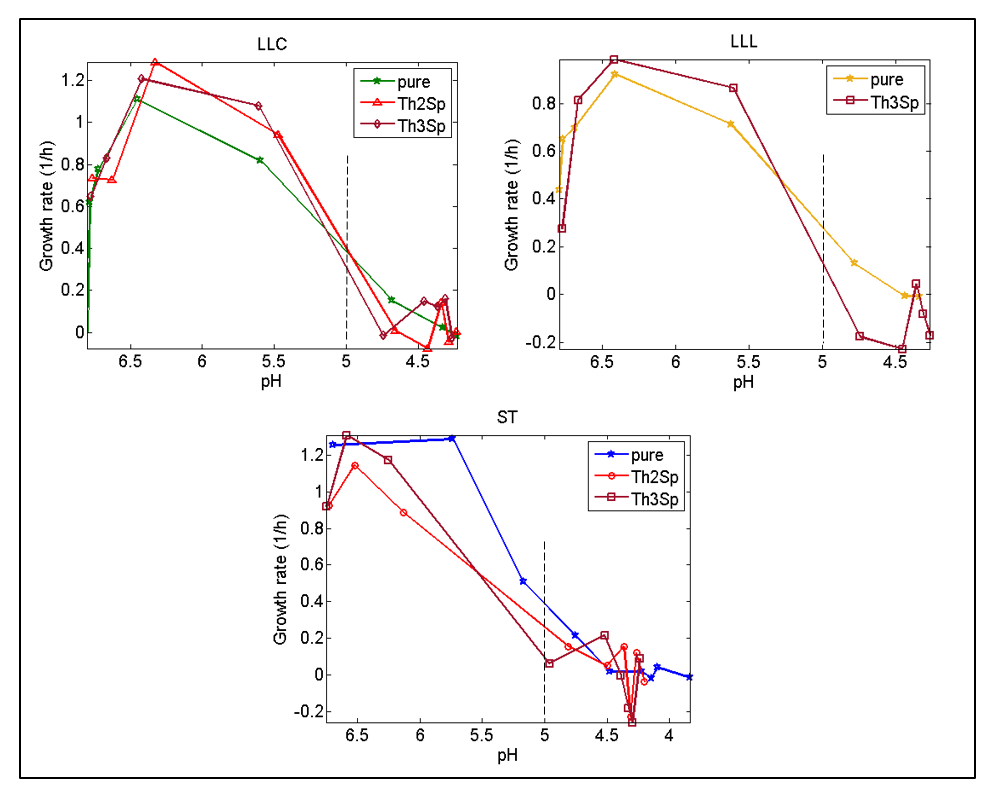


**Figure S2.** The growth performance of the *L. lactis* and *S. thermophilus* strains based on the experimental growth rate with respect to pH in pure and thermophilic co-cultures. Th2Sp and Th3Sp denote the two and three-species thermophilic co-cultures. *L. lactis* subsp. *cremoris* (LLC), *L. lactis* subsp. *lactis* (LLL) and *S. thermophilus* (ST).


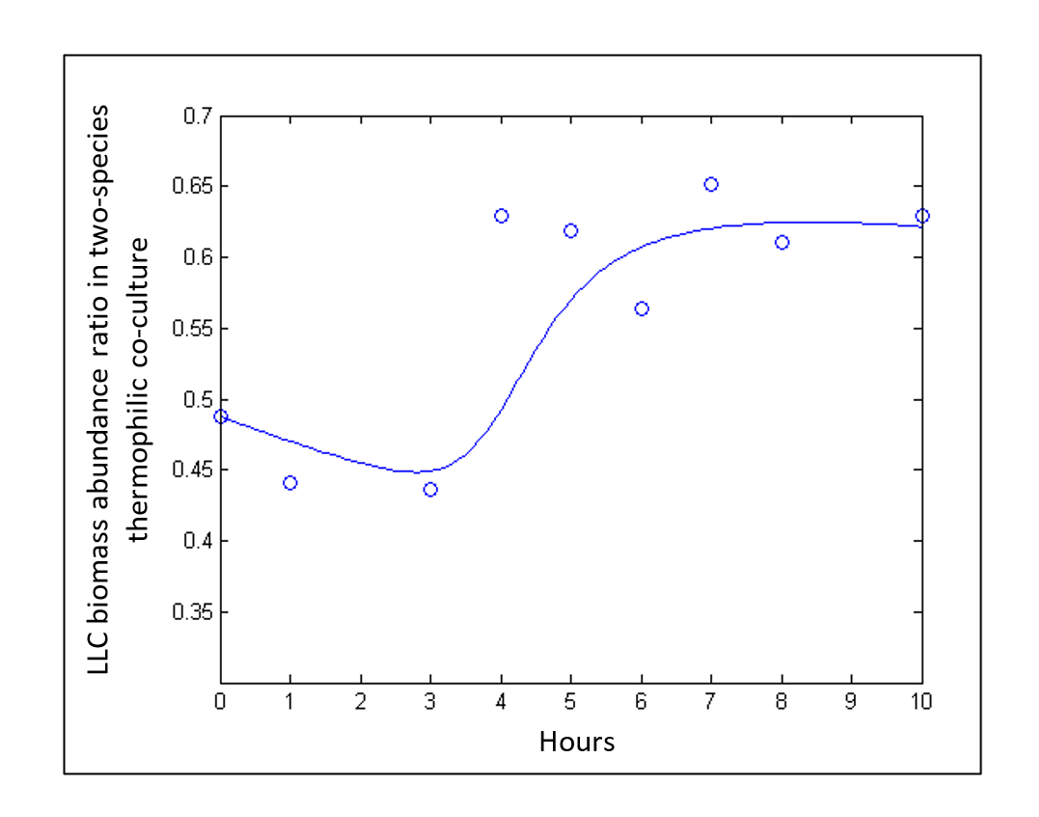


**Figure S3.** Individual biomass abundance ratio of *L. lactis* subsp. *cremoris* (LLC) in two-species thermophilic co-culture. Solid lines and points denote the model and average experimental results, respectively.


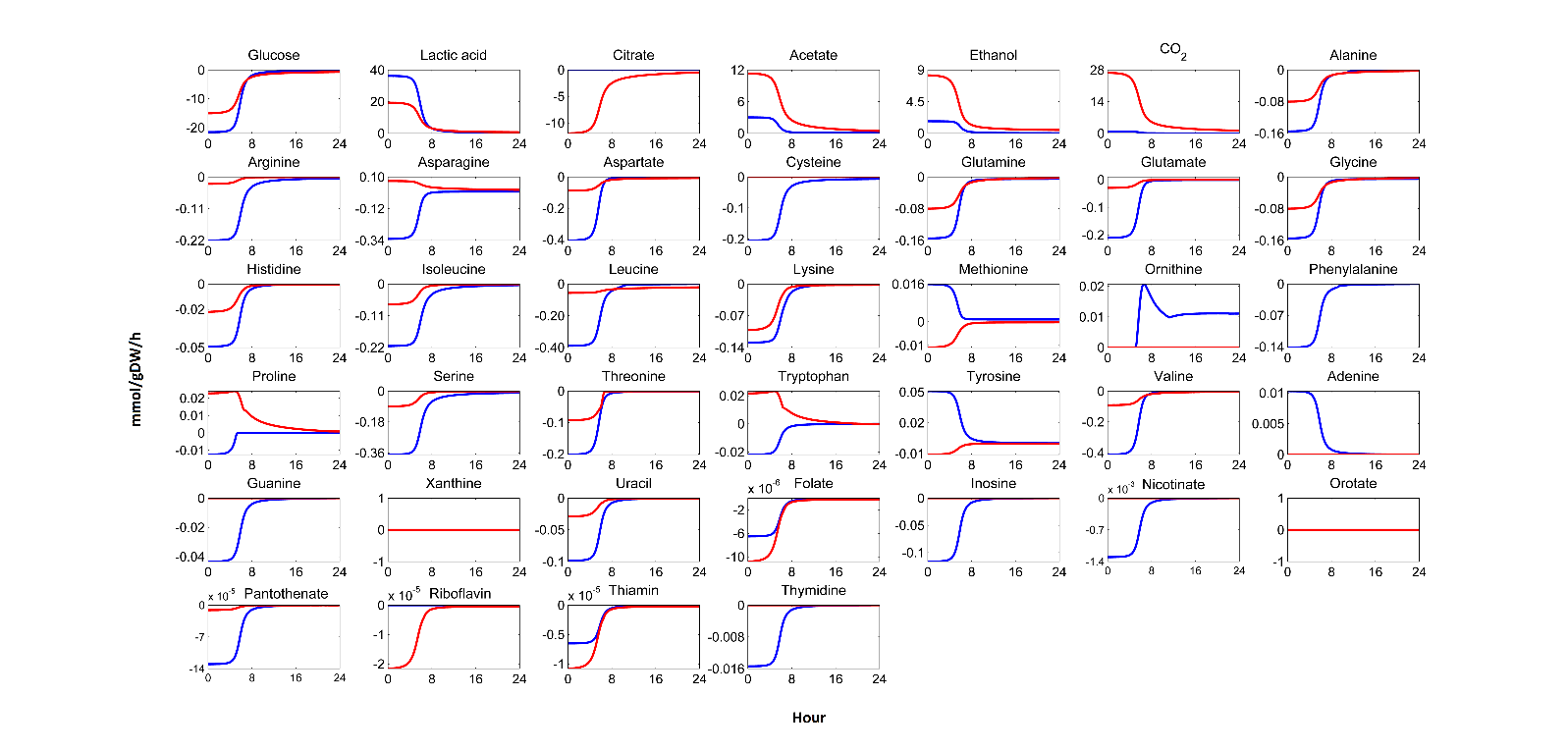


**Figure S4.** Individual exchange flux profiles of *L. lactis* subsp. *cremoris* (blue line) and *Leu. mesenteroides* (red line) in two-species mesophilic co-culture. Negative and positive flux values show consumption and production respectively.


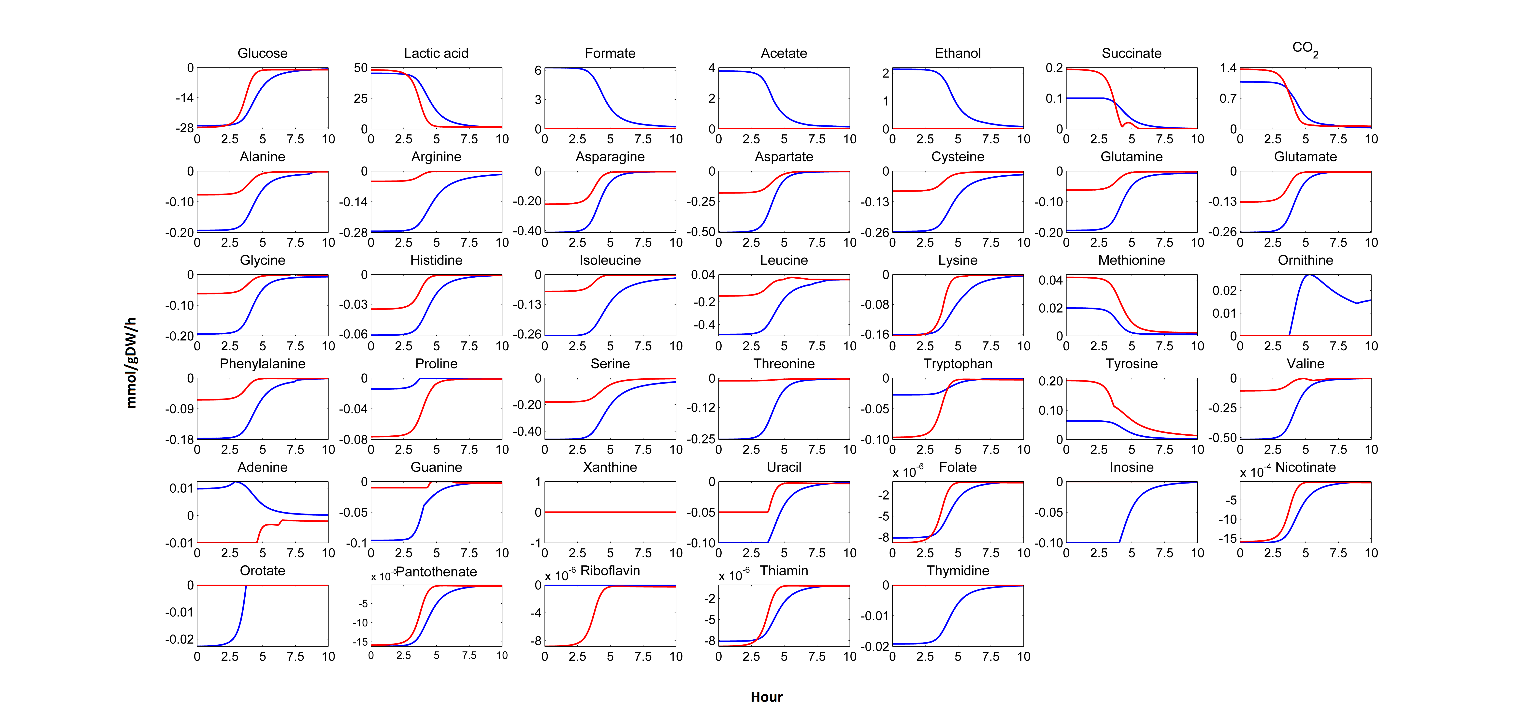


**Figure S5.** Individual exchange flux profiles of *L. lactis* subsp. *cremoris* (blue line) and *S. thermophilus* (red line) in two-species thermophilic co-culture. Negative and positive flux values show consumption and production respectively.


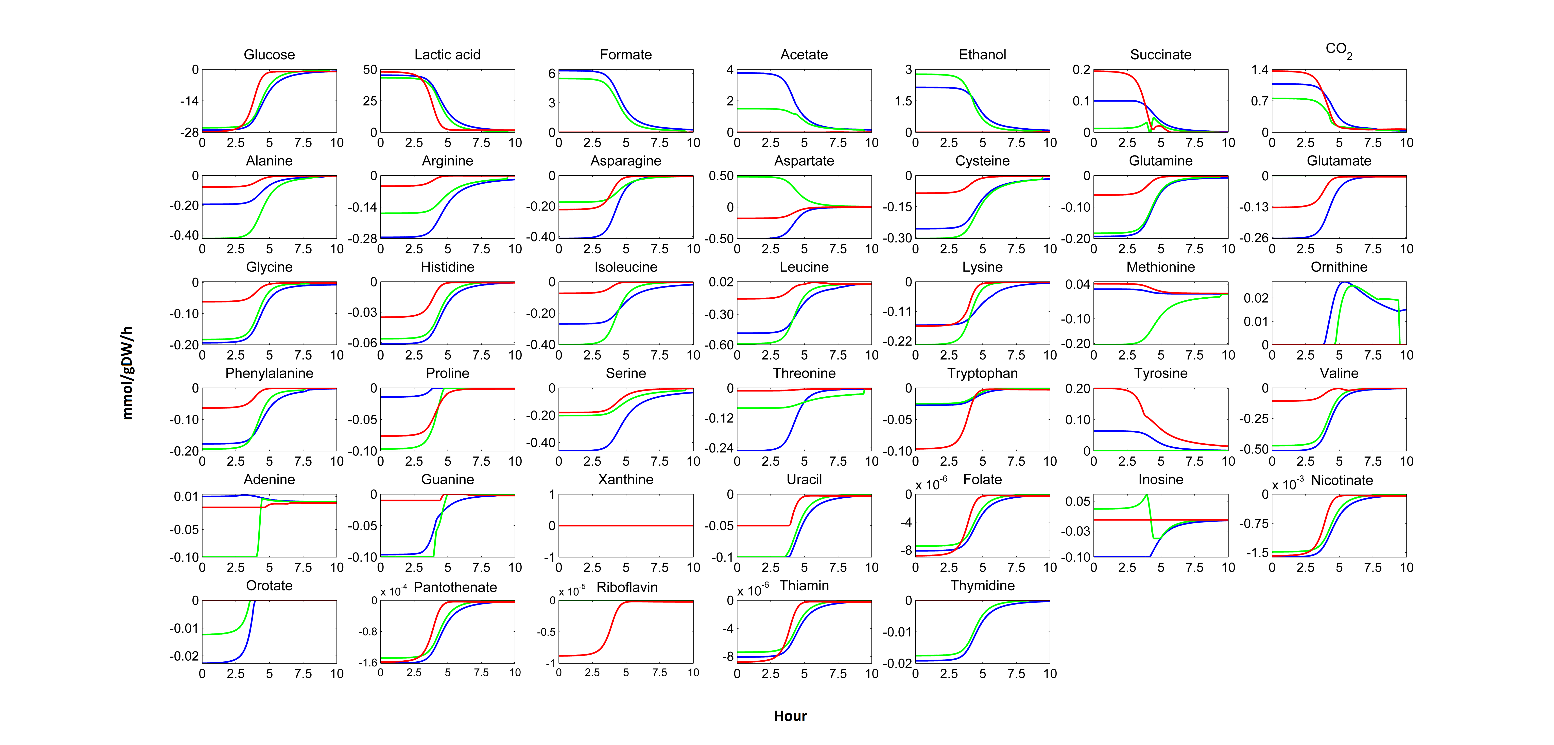


**Figure S6.** Individual exchange flux profiles of *L. lactis* subsp. *cremoris* (blue line), *L. lactis* subsp. *lactis* (green line) and S. thermophilus (red line) in three-species thermophilic co-culture. Negative and positive flux values show consumption and production respectively.
