# Supplementary material for: Dynamic co‐culture metabolic models reveal the fermentation dynamics, metabolic capacities and interplays of cheese starter cultures
Source: Biotechnol Bioeng. 2020 Sep 28;118(1):223–37. doi: 10.1002/bit.27565 (PMC7971941; doi:10.1002/bit.27565)
Supplement: Supplementary file 2 — Supporting information. [file BIT-118-223-s002.docx]

**Dynamic co-culture metabolic models reveal the fermentation dynamics, metabolic capacities and interplays of cheese starter cultures**

**Emrah Özcan^1,2^, Merve Seven^3^, Burcu Şirin^3^, Tunahan Çakır^4^, Emrah Nikerel^3^, Bas Teusink^1^* and Ebru Toksoy Öner^2^**

^1^ Systems Biology, Amsterdam Institute of Molecular and Life Sciences (AIMMS), VU Amsterdam, Amsterdam, The Netherlands

^2^ IBSB, Department of Bioengineering, Marmara University, Istanbul, Turkey

^3^ Genetics and Bioengineering Department, Yeditepe University, Istanbul, Turkey

^4^ Department of Bioengineering, Gebze Technical University, Gebze, Kocaeli, Turkey

* Corresponding author

### *Dynamic parameter estimation*

The parameters used in the substrate uptake kinetics were dynamically estimated using MEIGO optimization tool (Egea et al., 2014) in MATLAB, minimizing the optimization problem defined as follows:

$$\min_{p} J=\sum_{j=1}^{n} \sum_{i=1}^{t} (y_{j}(p,t_{i})-y_{ji}^{\text{experimental}})^{2}$$

subject to the system dynamics and parameter bounds:

$$\frac{dy_{i}}{dt}=V(y_{i},p)[biomass]$$

$$p_{i,\min}\leq p_{i}\leq p_{i,\max}$$

where n is the number of dependent variables (concentrations of biomass, glucose, lactic acid and amino acids), *t* is time, *y* is the matrix of dependent variables, *V* is reaction rates, and *p* is parameters. Reaction rates at each iteration of the algorithm were calculated by flux balance analysis. LSQNONLIN, which is a MATLAB algorithm solving non-linear least squares problems (The MathWorks^TM^), was used as the local solver in the optimization problem solved by MEIGO Toolbox.

***The parameters estimated***

In the substrate uptake kinetics defined in Eq. 4 of main text, *V_max_* and *V_min_* define the maximum and minimum utilization rate before and after the low pH conditions respectively, while *K_[LacH]_* defines the effect undissociated lactic acid on the rate, and the bigger *K_[LacH]_* is the earlier inhibition for the rate.

Before estimation of *V_max_*, *V_min_* and *K_[LacH]_* by the dynamic parameter estimation approach, the batch specific parameters, *C_1_* and *C_2_* in the substrate uptake kinetics were estimated by the non-linear regression of experimental [*LacH*] and [*Lac*] values using the Eq. 3 of the main text. Through the parameters *C_1_* and *C_2_*, the effect of batch specific pH profiles was also considered in the substrate uptake kinetics.

In dynamic parameter estimation, computational and experimental concentration values were compared, which minimizes the errors arising computational and experimental reaction rate comparison. Experimental concentration values are the direct results of bioanalyses such as HPLC, while experimental reaction rate values are calculated using these experimental concentration values but in relatively large time intervals, which might miss some dynamics for the system. The parameters estimated are listed in Table S1.

**Table S1.** The strain specific parameters used in the substrate uptake kinetics. The parameters were used in both pure and co-culture models. The unit of *V_max_* and *V_min_* are mmol/gDW/h, while the unit of *K_[LacH]_* is mmol^-1^. *L. lactis* subsp. *cremoris*, *L. lactis* subsp. *lactis*, *S. thermophilus* and *Leu. mesenteroides* are abbreviated as LLC, LLL, ST and LM, respectively.

|  |  | **LLC** | **LLL** | **ST** | **LM** |  |  | **LLC** | **LLL** | **ST** | **LM** |
| --- | --- | --- | --- | --- | --- | --- | --- | --- | --- | --- | --- |
| **Glc** | *V_max_* | 21.405 | 22.675 | 35.883 | 14.645 | ***Thr*** | *V_max_* | 0.200 | 0.066 | 0.010 | 0.087 |
|  | *K_[LacH]_* | 0.182 | 0.237 | 0.752 | 0.178 |  | *K_[LacH]_* | 0.272 | 0.063 | 0.250 | 0.138 |
|  | *V_min_* | 0.196 | 0.124 | 1.294 | 0.474 |  | *V_min_* | 0.002 | 0.001 | 0.004 | 0.006 |
| **Arg** | *V_max_* | 0.217 | 0.139 | 0.118 | 0.031 | ***Trp*** | *V_max_* | 0.048 | 0.078 | 0.138 | 0.060 |
|  | *K_[LacH]_* | 0.140 | 0.124 | 0.235 | 0.064 |  | *K_[LacH]_* | 0.210 | 0.500 | 0.358 | 0.052 |
|  | *V_min_* | 0.003 | 0.001 | 0.002 | 0.000 |  | *V_min_* | 0.003 | 0.001 | 0.007 | 0.002 |
| **Asn** | *V_max_* | 0.328 | 0.144 | 0.292 | -0.072 | ***Tyr*** | *V_max_* | -0.051 | 0.000 | -0.165 | 0.070 |
|  | *K_[LacH]_* | 0.280 | 0.183 | 0.591 | 0.083 |  | *K_[LacH]_* | 0.173 | 0.112 | 0.100 | 0.056 |
|  | *V_min_* | 0.002 | 0.001 | 0.006 | 0.000 |  | *V_min_* | 0.000 | -0.001 | 0.000 | 0.000 |
| **Asp** | *V_max_* | 0.403 | -0.400 | 0.240 | 0.083 | ***Val*** | *V_max_* | 0.410 | 0.393 | 0.200 | 0.092 |
|  | *K_[LacH]_* | 0.312 | 0.216 | 0.321 | 0.161 |  | *K_[LacH]_* | 0.260 | 0.323 | 0.100 | 0.139 |
|  | *V_min_* | 0.003 | -0.001 | 0.001 | 0.005 |  | *V_min_* | 0.003 | 0.001 | 0.004 | 0.000 |
| **Glu** | *V_max_* | 0.210 | 0.000 | 0.173 | 0.030 | ***Gln*** | *V_max_* | 0.150 | 0.150 | 0.080 | 0.080 |
|  | *K_[LacH]_* | 0.286 | 0.390 | 0.755 | 0.272 |  | *K_[LacH]_* | 0.240 | 0.241 | 0.500 | 0.160 |
|  | *V_min_* | 0.001 | -0.001 | 0.005 | 0.000 |  | *V_min_* | 0.005 | 0.003 | 0.003 | 0.001 |
| **Ile** | *V_max_* | 0.214 | 0.334 | 0.232 | 0.071 | ***Gly*** | *V_max_* | 0.150 | 0.150 | 0.080 | 0.080 |
|  | *K_[LacH]_* | 0.130 | 0.261 | 0.211 | 0.221 |  | *K_[LacH]_* | 0.240 | 0.340 | 0.500 | 0.160 |
|  | *V_min_* | 0.001 | 0.001 | 0.013 | 0.000 |  | *V_min_* | 0.005 | 0.003 | 0.003 | 0.001 |
| **Leu** | *V_max_* | 0.380 | 0.492 | 0.219 | 0.050 | ***Ala*** | *V_max_* | 0.150 | 0.350 | 0.100 | 0.080 |
|  | *K_[LacH]_* | 0.175 | 0.275 | 0.534 | 0.052 |  | *K_[LacH]_* | 0.200 | 0.200 | 0.400 | 0.160 |
|  | *V_min_* | 0.010 | 0.001 | 0.012 | 0.006 |  | *V_min_* | 0.005 | 0.003 | 0.003 | 0.001 |
| **Lys** | *V_max_* | 0.129 | 0.194 | 0.219 | 0.193 | ***Pro*** | *V_max_* | 0.150 | 0.150 | 0.100 | 0.080 |
|  | *K_[LacH]_* | 0.123 | 0.385 | 0.428 | 0.333 |  | *K_[LacH]_* | 0.200 | 0.200 | 0.400 | 0.160 |
|  | *V_min_* | 0.000 | 0.001 | 0.001 | 0.002 |  | *V_min_* | 0.005 | 0.003 | 0.002 | 0.001 |
| **Met** | *V_max_* | -0.015 | 0.170 | -0.053 | 0.010 | ***His*** | *V_max_* | 0.120 | 0.120 | 0.100 | 0.080 |
|  | *K_[LacH]_* | 0.450 | 0.151 | 0.265 | 0.294 |  | *K_[LacH]_* | 0.200 | 0.200 | 0.400 | 0.160 |
|  | *V_min_* | -0.001 | 0.001 | -0.003 | 0.001 |  | *V_min_* | 0.005 | 0.003 | 0.003 | 0.001 |
| **Phe** | *V_max_* | 0.141 | 0.157 | 0.116 | 0.065 | ***Cys*** | *V_max_* | 0.200 | 0.250 | 0.100 | 0.050 |
|  | *K_[LacH]_* | 0.180 | 0.321 | 0.229 | 0.227 |  | *K_[LacH]_* | 0.150 | 0.150 | 0.450 | 0.160 |
|  | *V_min_* | 0.001 | 0.005 | 0.001 | 0.000 |  | *V_min_* | 0.005 | 0.003 | 0.020 | 0.001 |
| **Ser** | *V_max_* | 0.364 | 0.168 | 0.238 | 0.090 | ***Cit*** | *V_max_* |  |  |  | 12.00 |
|  | *K_[LacH]_* | 0.135 | 0.122 | 0.210 | 0.217 |  | *K_[LacH]_* |  |  |  | 20.00 |
|  | *V_min_* | 0.006 | 0.001 | 0.003 | 0.000 |  | *V_min_* |  |  |  | 0.020 |
|  |  |  |  |  |  |  | *C_1_* | -0.112 | -0.124 | -0.147 | -0.105 |
|  |  |  |  |  |  |  | *C_2_* | 5.382 | 5.728 | 6.924 | 5.531 |

Since the chromatographic peaks of glutamine/glycine and alanine/proline pairs were overlapped, and also histidine and cysteine could not be detected by the current HPLC method used, these six amino acids were not considered in dynamic parameter estimation analysis. Since lack of the experimental evidence, the parameters used for the uptake kinetics of these six amino acids were chosen with the following criteria: A moderate value was chosen for the *K_[LacH]_* values which was in the range of *K_[LacH]_* values estimated by the dynamic parameter estimation analysis for the corresponding strain, while *V_max_* and *V_min_* values were chosen as much as minimum. All chosen parameters were aimed not to limit the in-silico growth.

Citrate was not consumed significantly by pure culture of *L. lactis* and S*. thermophilus* strains, while *Leu. mesenteroides* which is known as a citrate consumer lactic acid bacteria (Özcan et al., 2019; Smid and Kleerebezem, 2014) consumed all citrate before the stationary phase. Although the undissociated lactic acid was not the rate limiting compound for the citrate uptake rate in pure culture of *Leu. mesenteroides*, as the citrate was consumed before the undissociated lactic acid reached the rate limiting concentrations, citrate consumption by *Leu. mesenteroides* in the co-cultures was affected by acidic conditions. Hence, the substrate uptake kinetics defined in the main text was also used for citrate uptake, and the parameters used was estimated by manually fitting, changing the kinetic parameters until the simulation agreed most with the experimental data.

Co-culture specific parameters *C_1_* and *C_2_* (Table S2), which consider the co-culture specific pH profiles, were used in the co-culture models.

**Table S2.** Co-culture specific parameters, *C_1_* and *C_2_*

|  | Two species mesophilic co-culture | Three-species mesophilic co-culture | Two-species thermophilic co-culture | Three species thermophilic co-culture |
| --- | --- | --- | --- | --- |
| *C_1_* | -0.111 | -0.117 | -0.126 | -0.125 |
| *C_2_* | 5.397 | 5.540 | 5.999 | 5.916 |

**References**

Egea JA, Henriques D, Cokelaer T, Villaverde AF, MacNamara A, Danciu DP, Banga JR, Saez-Rodriguez J (2014) Meigo: An Open-Source Software Suite Based on Metaheuristics for Global Optimization in Systems Biology and Bioinformatics. BMC Bioinformatics 15:136

Özcan E, Selvi SS, Nikerel E, Teusink B, Toksoy Öner E, Çakır T (2019) A Genome-Scale Metabolic Network of the Aroma Bacterium Leuconostoc Mesenteroides Subsp. Cremoris. Applied Microbiology and Biotechnology

Smid EJ, Kleerebezem M (2014) Production of Aroma Compounds in Lactic Fermentations. Annual Review of Food Science and Technology, Vol 5 5:313-326
